# Optimized Multi-Stepped constant current constant voltage fast charging controller for lithium-ion batteries

**DOI:** 10.1038/s41598-025-25924-2

**Published:** 2025-11-18

**Authors:** Mohamed Magdy, Fatma Hanafy, Belal Abou-Zalam, Essam Nabil

**Affiliations:** 1 Department of Communication and Computer Engineering, Higher Institute of Engineering, El-Shorouk Academy, El-Shorouk City, Egypt; 2 Industrial Electronics and Control Engineerig Department, Faculty of Electronic Engineering, Menoufia University, Minouf, Egypt; 3 Technology of Biomedical Equipment Program, Faculty of Applied Health Sciences Technology, Menoufia University, Minouf, Egypt

**Keywords:** Lithium-ion battery, Fast charging, Constant current–constant voltage (CC–CV), Multi-stage charging controller, Particle Swarm optimization, Battery management system (BMS), Energy science and technology, Engineering

## Abstract

This paper addresses an effective, reliable and fast charging method for maximizing lithium-ion battery performance, longevity, and safety. The proposed multi-stage current charging mechanism utilizes a modified multi-stepped constant current-constant voltage based on the particle swarm optimization (MMSCC-CV-PSO) algorithm. The proposed MMSCC-CV-PSO charging strategy demonstrates a significantly faster charging performance compared to traditional charging techniques, including the widely used constant-current constant-voltage (CC–CV) method and the conventional multistage constant current (MSCC) approach. This enhanced efficiency highlights the superiority of the proposed method over existing solutions discussed in the literature. The simulation results confirm the practicality and superior performance of the proposed design strategy. Compared to the conventional MSCC method, the proposed approach achieved a considerable reduction in battery charging time, indicating a more efficient energy transfer process. Additionally, it led to a noticeable decrease in heat generation within the battery during the charging cycle, reflecting improved thermal management. These outcomes collectively demonstrate the effectiveness of the proposed method in enhancing both the speed and safety of the charging process, making it a promising solution for advanced battery management systems. The simulation model was developed and successfully implemented using the MATLAB/Simulink environment, providing a flexible platform for testing and evaluating the proposed charging strategy.

## Introduction

Lithium-ion batteries have emerged as the dominant energy storage solution across diverse applications, including portable electronics, electric vehicles, and renewable energy systems. Their widespread adoption stems from several superior characteristics: high energy density, extended cycle life, elevated working voltage, and absence of memory effects. Additional advantages such as cost-effectiveness, minimal self-discharge, environmental friendliness, and low self-consumption further contribute to their market dominance. These compelling benefits have positioned lithium-ion battery technology as a focal point in contemporary energy research and development^1,2^.

However, Lithium-ion battery technologies come with various concerns. One key issue is the need for efficient charging processes to enhance performance, extend lifespan, reduce charging times, minimize battery aging, and ensure reliable operation^3,4^. Another concern is related to the inescapable requirement, Due to the voltage and capacity limitations of a single cell. lithium-ion cell’s voltage is limited in the range of (2.4–4.2 V)^5^, To meet the demanding power and energy requirements of modern applications, battery systems are typically constructed using hundreds of individual cells connected in series or parallel configurations. These cells are combined to form battery modules, which are then integrated into complete battery packs^6–8^.

Hence, it demands to develop appropriate charging strategies. For instance, reducing battery-charging time while enhancing charging performance and prolonging battery life^9^. Additionally, these systems require ongoing monitoring and control to maintain optimal performance. This includes safeguarding against overcharging, over-discharging, and overheating, as well as managing the connection of cells in series to prolong their lifespan. The Battery Management System (BMS) is often referred to as the brain of the battery pack^10^.

**Fig. 1:**
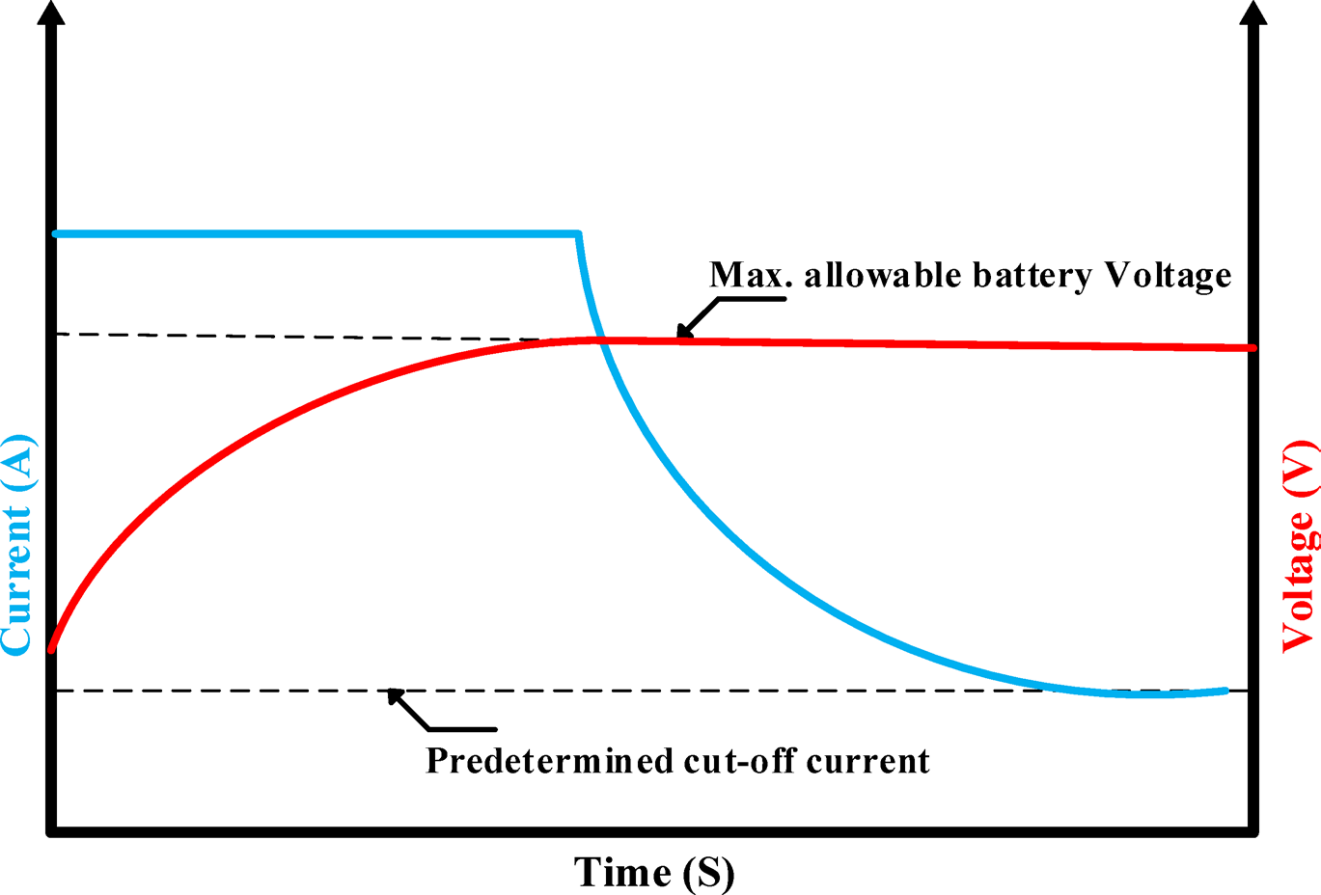
Fundamental of (CC–CV) charging technique.

Several works of literature have been published on charging methods of lithium-ion batteries. This work aims to enhance the time required for charging, rise in temperature, loss of energy, aging, and lifespan^3^. There are several ways to charge batteries, but the standard charging technique for lithium-ion batteries is constant current constant voltage (CC–CV)^11^. The (CC–CV) charging technique is widely recognized as the most widely utilized charging method for electronic items due to its ease of use and simplicity^12,13^. Figure 1 demonstrates the fundamentals of (CC–CV) charging technique which consist of two steps to this charging process. During the first stage of charging, a constant current (CC) is applied to the lithium-ion battery until the maximum voltage is reached, which is typically set at 4.2 V for Li-ion batteries. The second step of charging involves applying a constant voltage (CV) to the lithium-ion battery until the current hits the predetermined cut-off current value, which is usually 5% of the nominal current. At this point, the battery becomes fully charged^11^, While the traditional (CC–CV) charging method are more economical and easier to implement, it is incompatible with fast charging applications because it causes a striking temperature to increase in the constant current mode, and an extended charging time in the constant voltage mode which reducing battery life^12,14^.

Several charging techniques have been developed in the literature to address the drawbacks of the traditional (CC–CV) method. One of these techniques is the Multi-Stepped Constant Current (MSCC) method. Multi-stage charging has been found to minimize temperature rise while charging, which is important for keeping battery safety and health. It has also been demonstrated that MSCC charging techniques reduce the charging period when compared to typical constant current constant voltage (CC–CV) approaches^11^. The Multi-Stepped fast charging approach, which is motivated by physical degradation mechanisms, increases the batteries’ cycling lifetime by more than 200 full equivalent cycles (FEC), according to experimental results^15,16^. The design of a non-isolated Bidirectional DC/DC converter for lithium-ion battery charging will be thoroughly examined. The main contributions of the paper are summarized as follows:

1. This study presents the design of a non-isolated Bidirectional DC/DC converter for lithium-ion battery charging.
2. Create intelligent charging control to facilitate rapid charging with a suitable battery management system that keeps monitor on each cell’s temperature, voltage, and current in order to increase the longevity and safety of lithium-ion batteries.
3. Comparisons of results with the traditional (CC–CV) method and MSCC method were presented.

The structure of this research includes a literature review and state of the art, which is presented in section “Related work”. Section “System design considerations and methodology” highlights the system design considerations and methodology. In section “Proposed multi-stepped charging controller design”, the proposed multi-stepped charging controller design is presented. Section “Simulation methodology and comparative analysis” provide simulation results and discussion which presents the justification set up of the proposed design algorithm and performance comparison to existing methods. Finally, Section “Conclusion and future work” provides a concise summary of the findings, their implications, and suggestions for future research in advance.

## Related work

Several researchers have proposed multi-stage constant current (MSCC) charging strategies to shorten charging times and reduce temperature rise during the charging of lithium-ion batteries. For instance, Jinlei Sun^17^ introduced an MSCC optimization strategy incorporating lithium plating detection using electrochemical impedance spectroscopy (EIS) analysis. Compared to the traditional constant current–constant voltage (CC–CV) method, this approach reduced charging time by 21.44% and energy consumption by 15.7%, while enabling real-time monitoring of lithium plating—thereby enhancing both safety and efficiency during fast charging. Similarly, Tahir et al.^16^ presented a novel approach to addressing the key challenge of fast charging in electric vehicles (EVs) by optimizing the charging strategy for lithium-ion batteries (LIBs) using the Taguchi method for multi-objective optimization of the multi-stage constant current. the experimental results show that, at 25 °C, the multi-objective optimization of the multi-stage constant current charging profile enables the LIB to charge faster 12% than CCCV. Sang-Sun Yun^19^. introduced a multilevel multistage constant-current constant-voltage (MLMCC-CV) superfast charging technique for batteries, which adjusts the charge voltage according to the charge current The technique aims to prevent overvoltage protection activation in the battery management system (BMS) due to transient responses caused by capacity deviations between multiple cells during charging, adjusts voltage based on current to avoid these interruptions. Though it’s about 3% slower in charging rate compared to older methods, it offers a smoother, safer charge without early shutdowns.

In^20^ Sabarimuthu, introduced the MSCC-CV-CT charging method, which emphasizes controlling cell temperature as a key element in battery charging dynamics. The performance of electric vehicles and the lifespan of lithium-ion batteries may both be enhanced by this study’s noteworthy development in battery charging technology. Mochamad Ari Bagus Nugroho^21^ developed the MCC-CV method as an enhancement over the conventional CC–CV (Constant Current–Constant Voltage) charging method. The M CC–CV method increases initial charging currents by about 13% over the conventional CC–CV maximum, leading to a decrease in total charging duration of approximately 16% as demonstrated in experimental results. This translates to faster charging times while maintaining safety and battery health. Kartik Kumar^22^ presented an innovative approach to improve lithium-ion battery charging efficiency and lifespan through the optimization of a multistage constant current (5S-CC) charging method. The study employs Grey relational analysis (GRA) combined with Taguchi Design of Experiment to determine the optimal current levels across five distinct stages, aiming to minimize charging time while controlling temperature and heat generation. The optimized 5S-CC method achieved a significant reduction in charging duration, reaching full capacity in approximately 48.11 min, compared to 73.48 min required by the conventional CC–CV_0.05C method, as verified through experimental results.

While methods such as the Taguchi method and Grey relational analysis have been applied to optimize MSCC charging profiles, they are not inherently designed for controller parameter optimization. In contrast, Ran Li^23^ presented the Particle Swarm Optimization (PSO) as a population-based metaheuristic algorithm that has been widely adopted for PID tuning in nonlinear systems due to its strong global search capability, fast convergence, and relatively low computational complexity. Recent studies have shown that PSO can significantly improve the efficiency and thermal safety of Li-ion charging strategies, and Mónica Camas-Náfate^24^ has also been demonstrated as an effective tool for battery modeling and parameter identification compared to other algorithms such as the Grey Wolf Optimizer. These findings support the choice of PSO in this work for optimizing PID parameters in advanced charging control.

Recent advances in battery management technologies have expanded beyond traditional CC–CV and MSCC approaches. Guo et al.^25^ introduced a data-driven electrode aging assessment framework for efficient sorting of retired batteries, enhancing prediction of degradation and second-life utilization. Guo and Shen^26^ evaluated battery peak power under various operational modes, highlighting the effects of different charge/discharge strategies on performance and safety. Xu et al.^27^ developed an optimization-driven active cell balancing strategy to mitigate cell inconsistency and extend operational time in energy storage systems. Additionally, Guo and Shen^28^ provided a comprehensive overview of state-of-power estimation methods, including error source analysis and strategies for improved battery efficiency and safety Tang et al.^29^ investigated pulse charging currents for lithium-ion batteries in low-temperature environments, demonstrating that optimized bi-directional and positive pulse currents can significantly reduce polarization and improve temperature control compared to conventional constant-current charging. Huang et al.^30^ proposed a negative pulsed charging strategy using NSGA-II optimization, which enhances charging efficiency and battery capacity while mitigating adverse thermal effects through electrothermal coupling simulations. Sinusoidal charging methods have also gained attention. Huang et al.^31^ evaluated a CC-sinusoidal reflex charging technique, showing reduced electrode degradation and improved stability of LiCoO_2_ and graphite electrodes over multiple cycles^3^. Integrating these studies establishes a robust context for the proposed MMSCC–CV–PSO strategy, demonstrating its relevance and novelty within modern battery management research. The proposed MMSCC-CV-PSO strategy offers a complementary approach, combining multi-stage current profiles with PSO-tuned PID control for precise current regulation, balancing fast charging performance with battery safety and long-term reliability.

In this paper, a modified multi-stepped constant current-constant voltage based on the particle swarm optimization (MMSCC-CV-PSO) algorithm is proposed. It can be used to minimize charging time with decrease heat generation while battery charging while maintaining safety, battery health and improving performance.

## System design considerations and methodology

### Battery specifications

The experimental system utilizes an LG INR18650 B4 lithium-ion cell as the fundamental electrochemical storage unit. A high-capacity battery pack was constructed through a 7-series, 19-parallel (7S19P) configuration to meet the voltage and current requirements of the study. The cell specifications are characterized provided below in Table 1^32^.

**Table 1:** Technical specifications of the lithium-ion battery cell.

Parameter	Value
Cell model	18,650 Lithium-Ion INR18650 B4 2600 mAh
Form	Cylindrical cell
Cathode chemistry	Lithium ion, NMC (Nickel, Manganese and Cobalt)
Nominal voltage	3.6 V
Nominal capacity	2.6 Ah
Standard charging protocol	CC–CV @0.5C (1250 mA), 4.2 V, 4.20 ± 0.05 V max, 1.0 C (2500 mA) max, 0.02C (50 mA) cut-off
Standard discharging protocol	CC@0.2 C (500 mA), 2.75 V cut-off
Operating temperature	Charging: 0 to 45 °C Discharging: − 20 °C to + 60 °C
Internal impedance	70
Mass	48 g

The battery model was implemented in MATLAB/Simulink using the Lithium-Ion Battery block from Simscape Electrical. All parameters were set according to the manufacturer’s datasheet for the INR18650 B4 (2600 mAh) lithium-ion cell, including nominal voltage, rated capacity, internal resistance, and thermal properties. The OCV–SoC curve was derived from manufacturer data and incorporated into the model. Heat generation is computed internally within the Simulink block based on electrical losses and the specified thermal capacity. This ensures that the simulated cell behavior closely matches the actual performance of the real battery^32^.

### Non-isolated bidirectional DC–DC converter

**Fig. 2:**
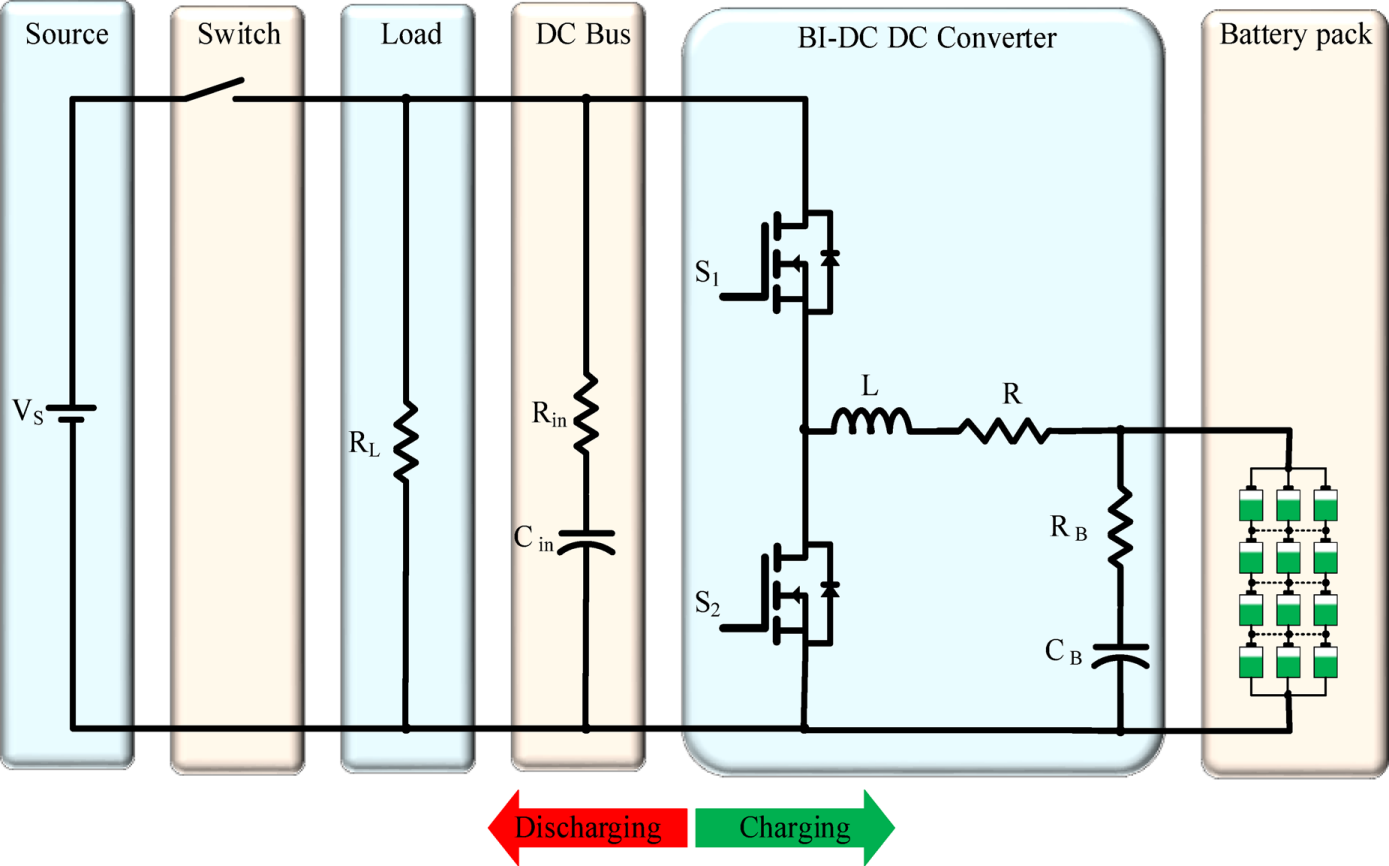
Fundamental topologies of non-isolated bidirectional DC/DC converters.

To execute all operations efficiently, a selection of highly effective power electronic devices is essential. The most commonly utilized DC/DC converters can be categorized into isolated and non-isolated types^33^. The study focuses on a non-isolated bi-directional DC/DC converter^34^. Figure 2 shows the fundamental topology of non-isolated Bi DC/DC In this setup, a battery pack is connected to a bi-directional DC/DC converter, which is subsequently linked to a load. This selection is motivated by the need for energy transfer in both directions. The non-isolated bi-directional DC/DC converter is chosen for its compact design, lightweight nature, and high efficiency, making it particularly suitable for various applications involving lithium-ion batteries^34,35^.The main required parameters for Non-isolated Bidirectional DC–DC Converter determined by equations in Table 2^36^. Table 3 presents the technical specifications of the proposed system architecture, quantifying the key design parameters and performance characteristics of the implemented solution^13^.

**Table 2:** Design parameters for non-isolated bidirectional DC–DC converters.

Buck	Boost	Parameter
Duty cycle calculation		
${D}_{(buck)}=\frac{{v}_{out}}{{v}_{in\left(max\right)}\times \eta }$	${D}_{(boost)}=1-\frac{{v}_{in\left(min\right)}\times \eta }{{v}_{out}}$	Where ${v}_{in\left(max\right)}=\text{maximum input voltage}$ ${v}_{in\left(min\right)}=\text{minimum input voltage}$ ${v}_{out}=\text{ desired output voltage}$ ${D}_{(buck)}=\text{minimum duty cycle for buck mode}$ ${D}_{\left(boost\right)}=\text{maximum duty cycle for boost mode}$ $\eta =\text{estimated efficiency at calculated }{v}_{in},{v}_{out},\text{ and }{I}_{out}$
Inductor selection		
$L>\frac{{v}_{out}\times \left({v}_{in\left(max\right)}-{v}_{out}\right)}{{K}_{ind}\times {F}_{sw}\times {I}_{out}\times {v}_{in\left(max\right)}}$	$L>\frac{{{v}_{in\left(min\right)}}^{2}\times \left({v}_{out}-{v}_{in\left(min\right)}\right)}{{K}_{ind}\times {F}_{sw}\times {I}_{out}\times {{v}_{out}}^{2}}$	${v}_{in\left(max\right)}=\text{maximum input voltage}$ ${v}_{in\left(min\right)}=\text{minimum input voltage}$ ${v}_{out}=\text{ desired output voltage}$ ${I}_{out}=desired \;maximum \;output \;current$ ${F}_{sw}=switching \;frequency \;of \;the \;converter$ ${K}_{ind}=estimated \;coefficient \;that \;represents$ $the amount \;of \;inductor\; ripple \;current \;relative$ $to \;the \;maximum \;output$ $current$
Output capacitor selection		
${C}_{out}=\frac{{K}_{ind}\times {I}_{out}}{8\times {F}_{sw}\times {\Delta v}_{out}}$	${C}_{out}=\frac{{D}_{(boost)}\times {I}_{out}}{{F}_{sw}\times {\Delta v}_{out}}$	Where ${v}_{in\left(max\right)}=\text{maximum input voltage}$ ${v}_{in\left(min\right)}=\text{minimum input voltage}$ ${v}_{out}=\text{ desired output voltage}$ ${\Delta v}_{out}=\text{ desired output voltage ripple}$ ${D}_{\left(boost\right)}=\text{maximumduty cycle for boost mode}$ ${I}_{out}=desired \;maximum \;output \;current$ ${F}_{sw}=switching \;frequency \;of\; the\; converter$

**Table 3:** Specification of the system parameter.

Parameter	Value	Unit
$v_{{out\left( {min} \right)}}$	25.2	$V$
$v_{{out\left( {max} \right)}}$	29.4	$V$
$I_{out} \left( {max} \right)$	76.5	$A$
$v_{{in\left( {min} \right)}}$	45	$V$
$v_{{in\left( {max} \right)}}$	48	V
$Switching\;frequency$	62.5	kHz
$V_{ripple}$	0.1	V
$\eta$	90% : 95%	–
$L$	0.576	mH
$R$	6	Ω
$C$	1000	µF

### Conventional multi-stepped constant current (MSCC) charging

**Fig. 3:**
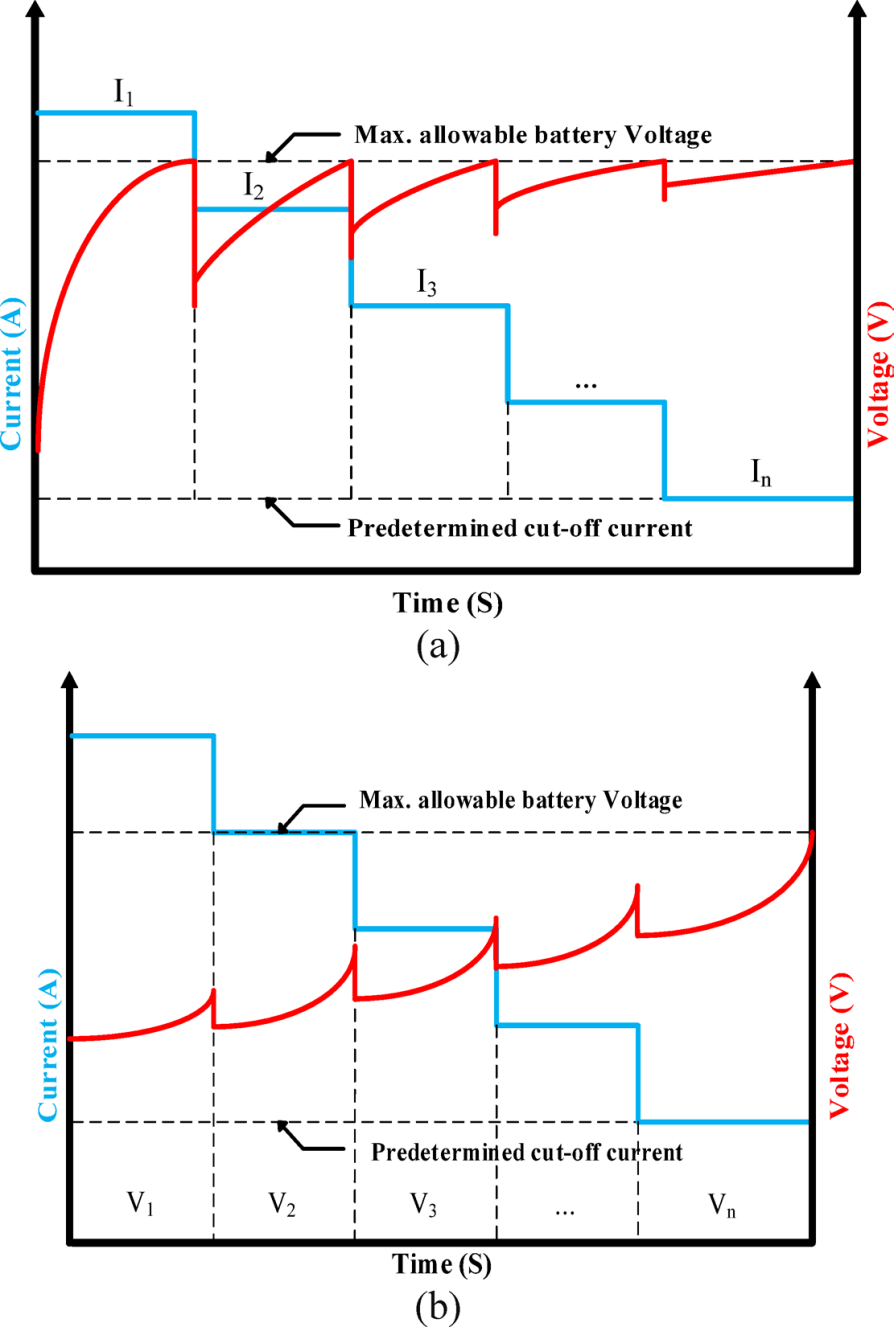
The MSCC voltage-based strategies charging technique: (**a**) Cut-off voltage-based criteria. (**b**) Voltage threshold crossing-based criteria.

MSCC charging employs a stepwise current reduction strategy, where the charging process is divided into multiple discrete constant-current phases with progressively decreasing magnitudes with the design specifications of battery charging^18^. The initial charging phase employs elevated current levels, which are systematically reduced in subsequent stages as the battery approaches full charge capacity. MSCC charging implementations predominantly utilize two distinct control approaches: voltage-dependent and state-of-charge (SoC)-dependent strategies. Among these, voltage-based methods offer distinct advantages as they maintain consistent operational thresholds regardless of battery capacity fade, eliminating the need for periodic recalibration^11,37,38^. Stage transition mechanisms in voltage-based approaches are implemented through either fixed Cut-off Voltage Termination or dynamic Voltage Threshold Crossing as shown in Fig. 3a and b respectively. In addition, the critical design parameters for MSCC systems include stage quantity optimization, current magnitude selection, and phase duration criteria.

One of the most important research problems in battery management systems is creating the ideal multi-stage constant-current (MSCC) charging profile. To create optimal current-stage configurations, scholarly research has used a variety of methodological techniques that can be broadly divided into three paradigms, which are empirical techniques, computational optimization, and analytical modeling^11^. The optimal charging pattern by Claude Ziad El-Bayeh^39^ used analytical methodologies and applied to determine the optimal charging current rates are calculated numerically using Eq. (1) and subsequently verified through experimental testing.

1 $${I}_{n}=\sqrt{ {I}_{n-1}*{I}_{n+1}    {I}_{n}}>3$$

In this case, ${I}_{n}$ indicates the current at stage *n*, where ${I}_{n-1}$ represents the preceding stage’s current and ${I}_{n+1}$ corresponds to the following stage’s current. Equation (1) is specifically applied when the battery manufacturer specifies both the initial and final charging currents and the total number of charging stages is an odd integer. This approach ensures systematic current reduction while maintaining operational constraints.

## Proposed multi-stepped charging controller design

**Fig. 4:**
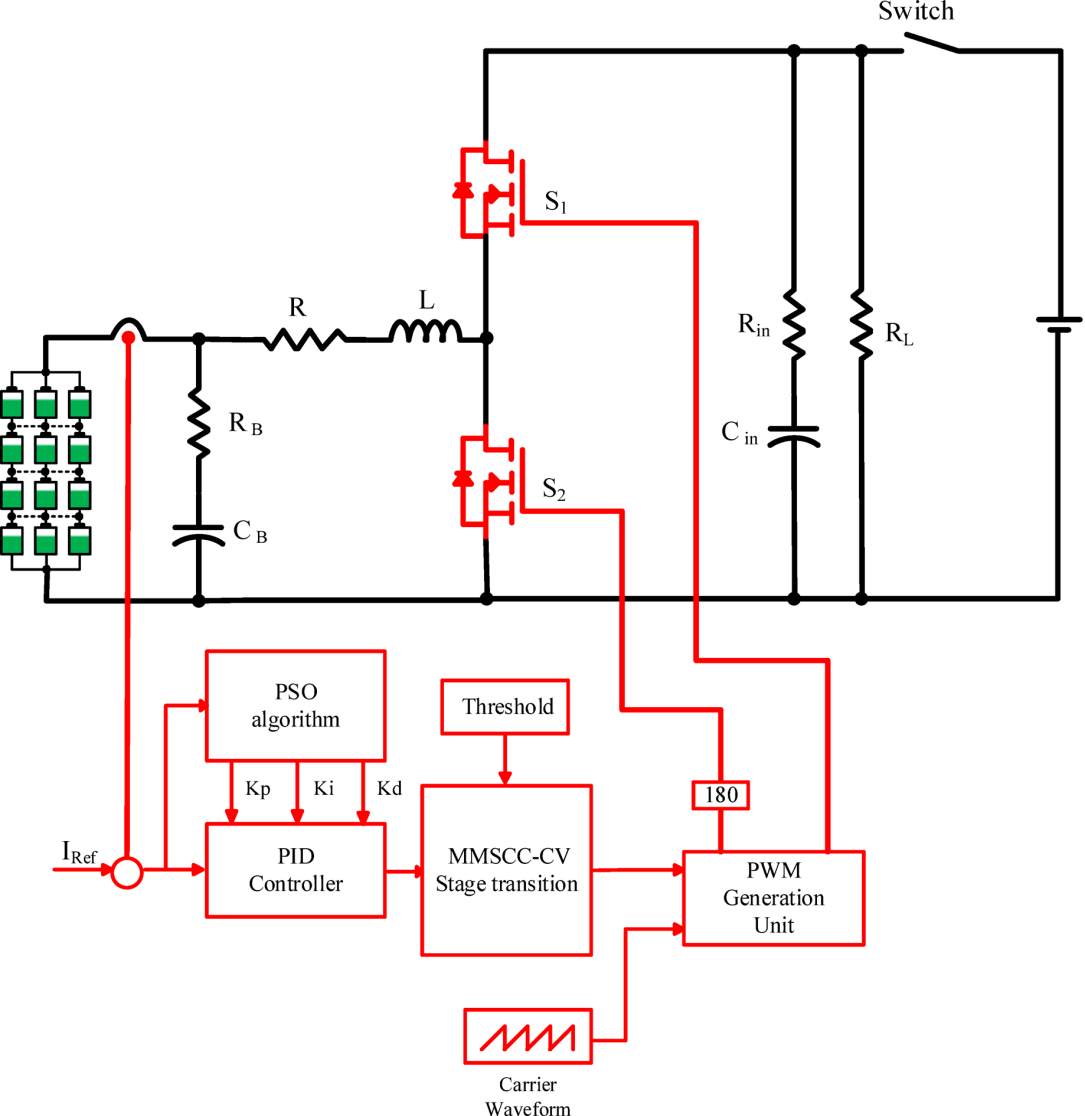
The proposed battery charging methodology, along with the control architecture of the presented system utilizing a PID controller.

The proposed modified multi-stepped constant current-constant voltage based on the particle swarm optimization (MMSCC-CV-PSO) algorithm enhances conventional MSCC charging through modifying the traditional MSCC charging method by using two constant-current (CC) phases followed by a constant-voltage (CV) phase, addressing the critical limitation of full charging of battery and low heat generation in traditional multistage charging systems. As illustrated in Fig. 4, the architecture integrates charging and discharging module which dynamically adjusts switching based on pulse width modulation output from controller and current controller which modifies CC stage duration via voltage thresholds.

### Control algorithm

**Fig. 5:**
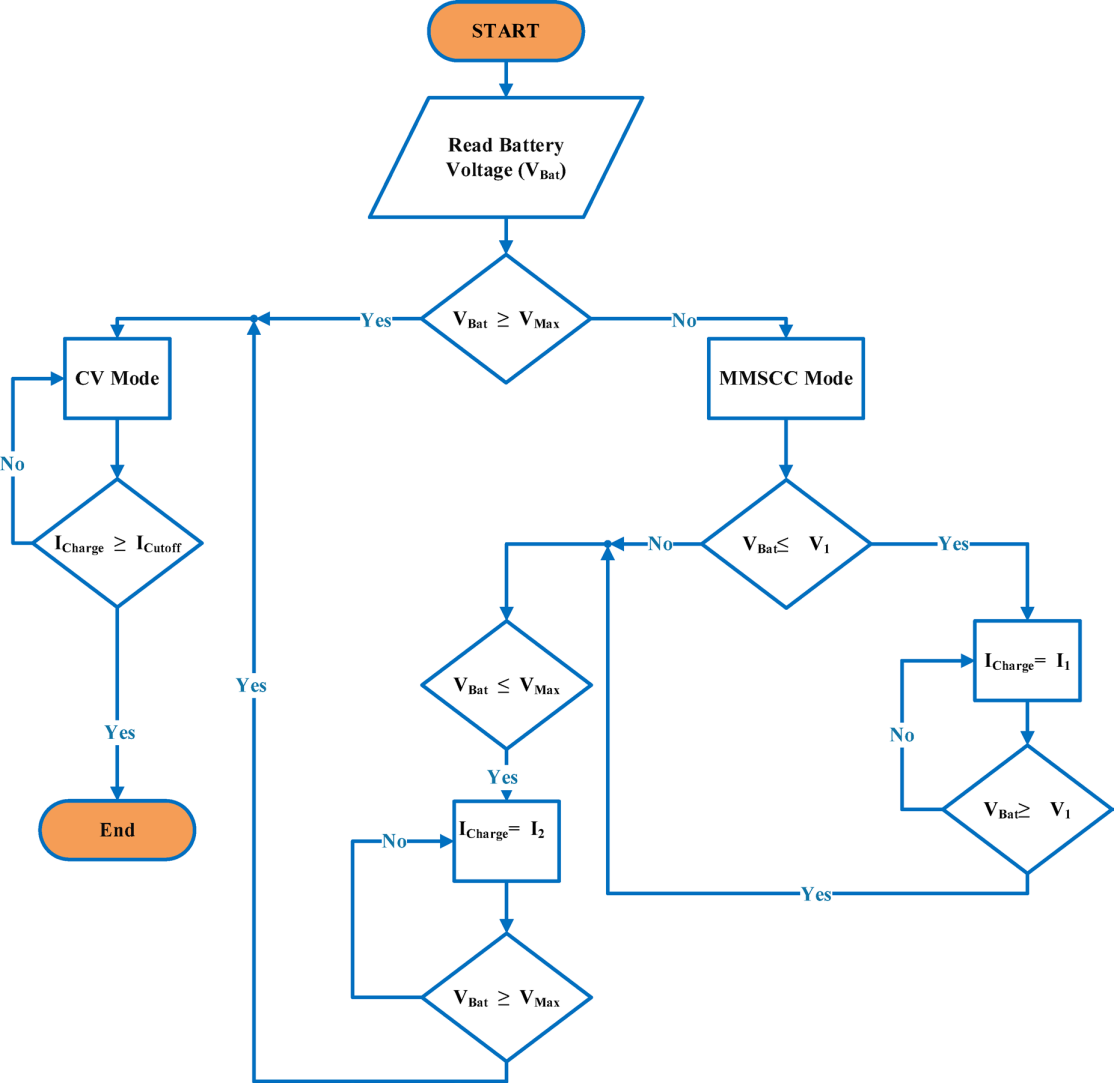
Illustrates the charging control algorithm for the proposed MMSCC-CV-PSO battery charging method.

The charging technique follows an established sequence, as shown in Fig. 5. Starting with the Initialization Phase, the optimized MSCC profile is used to apply the first Constant Current (CC) charging current, ${I}_{peak}$. This keeps happening until the first threshold voltage, $\left({V}_{1}\right)$, is reached. This voltage stays below the cutoff voltage of the battery. This is followed by the Normal Phase, which involves applying a charging current ${I}_{charge}$ until the battery reaches the second threshold value (cutoff voltage) $\left({V}_{\text{max}}\right)$. Ultimately, the protocol switches to a steady voltage charging technique during the Full Charge Phase, which lasts until the battery is completely charged. This systematic methodology ensures charging process efficiency and safety.

### Phase transition logic and waveform characteristics

**Fig. 6:**
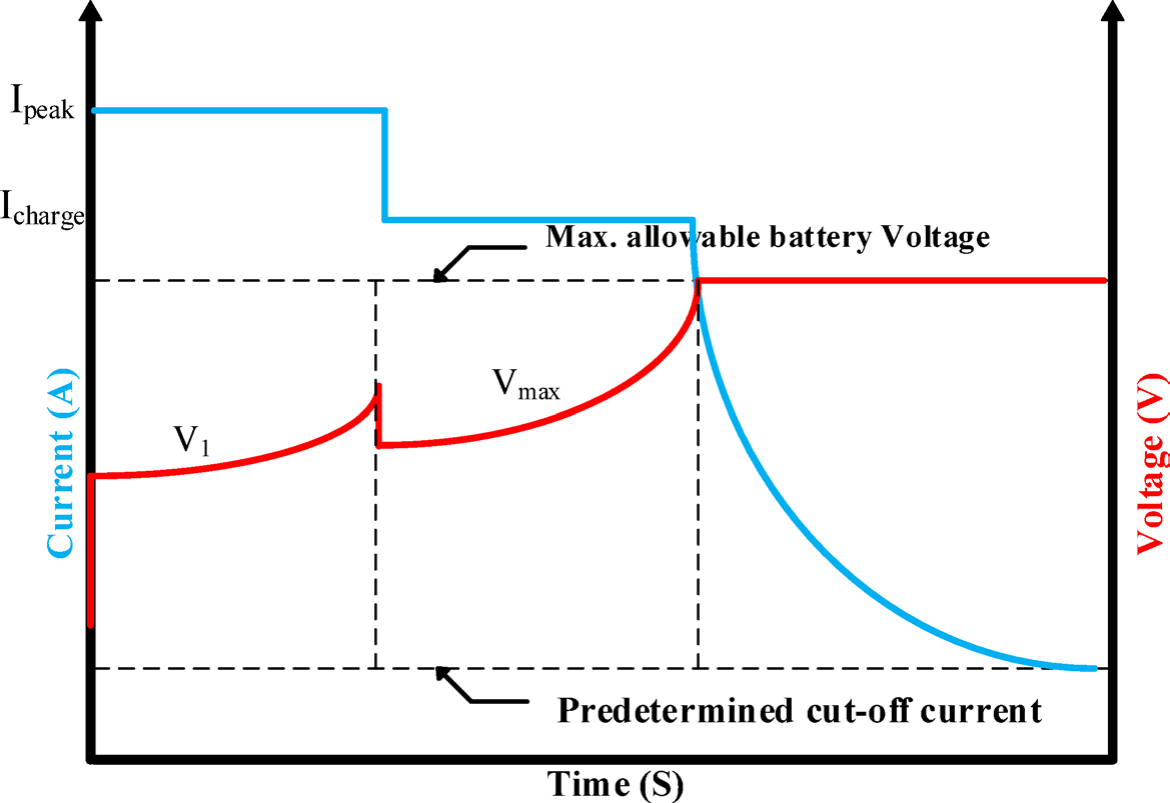
Waveform characteristics.

According to the charging procedure, when the battery voltage, ${(\text{V}}_{\text{battery}})$, approaches or exceeds the threshold voltage, ${(\text{V}}_{\text{threshold}})$, the current Constant Current (CC) stage should be stopped. Using the currents (${\text{I}}_{\text{peak}})$ and ${(\text{I}}_{\text{charge}})$, the process then moves on to the following CC step, which continues until the battery is fully charged. The current and voltage characteristics during the three stages of the (MMSCC-CV-PSO) charging process are provided in Table 4. The waveform features depicted in Fig. 6 demonstrate that the current profile maintains the stepped current structure that is different of the MSCC methodology. In the mean time, the voltage envelope shows clear threshold values that mark important charging process transitions. This thorough examination highlights how well the technique controls voltage and current during the charging stages.

**Table 4:** current and voltage of three phases of (MMSCC-CV-PSO) charging method.

Phase	Cell voltage	Total pack voltage	Current per cell	Total pack current
cc	4.1	28.7	4A (1.6C)	76 A
cc	4.2	29.4	2.5A (1C)	47.5 A
cv	4.2	29.4	2.5A (1C) to 0.1A (0.04C)	47.5 A to 1.9 A

### Optimized charging strategy: multi-stage approach

To improve charging efficiency and extend battery lifespan, this study implements the MMSCC-CV-PSO charging strategy is structured into two sequential constant-current (CC) phases followed by a constant-voltage (CV) stage, as shown in Fig. 6. In the first CC phase, the battery is charged with the peak current ${(\text{I}}_{\text{peak}})$, representing the maximum current the battery can safely tolerate according to the manufacturer’s datasheet. During the second CC phase, a normal current ${(\text{I}}_{\text{charge}})$, corresponding to the normal charging current provided in the datasheet, is applied to continue charging efficiently while maintaining safe operation. The voltage threshold $\left({V}_{1}\right)$, controlling the transition between the two CC phases is defined as a design parameter to split the constant-current stage, allowing the initial high current to accelerate charging while the subsequent lower current reduces heat buildup before reaching the maximum battery voltage $\left({V}_{\text{max}}\right)$. These key parameters (${\text{I}}_{\text{peak}}$, ${\text{I}}_{\text{charge}}$, and ${V}_{1}$) are fixed based on battery specifications and design considerations and are not part of the PSO optimization, which is exclusively used to fine-tune the PID controller gains (Kp, Ki, Kd) for optimal dynamic response. This strategy ensures a safe, efficient, and reproducible charging profile, effectively balancing charging speed, thermal management, and long-term battery reliability. In addition to the multi-stage constant current (MSCC) and constant voltage (CV) charging phases, advanced energy management strategies are employed to enhance battery longevity and operational efficiency. Specifically, adaptive thermal and power management techniques adjust the charging rate dynamically according to battery state-of-health (SoH), depth-of-discharge (DoD), and temperature. By continuously monitoring these parameters, the system optimizes the charging current and prevents excessive thermal stress, which can otherwise accelerate battery degradation. For hybrid energy storage systems incorporating supercapacitors, this adaptive approach further ensures balanced energy distribution, mitigates deep discharge of the batteries, and increases the overall energy throughput. Such optimization methods have been shown to extend battery lifetime, improve safety, and provide environmentally and economically beneficial management of lithium-based energy storage systems^40,41^.

### Advantages

Significant advantages are provided by the (MMSCC-CV-PSO) approach in terms of dynamic performance and lifetime extension. Notably, it reduces heat generation stress in comparison to the traditional MSCC, which is important for increasing the battery’s longevity and dependability. Furthermore, the (MMSCC-CV-PSO) method offers a minor advantage over conventional approaches in terms of dynamic performance, reaching 100% State-of-Charge (SOC). It also excels in speed, charging more quickly than the CC–CV technique. The (MMSCC-CV-PSO)’s ability to maximize battery management and performance is demonstrated by these benefits.

### PSO-PID optimization pseudocode

PSO is a population-based stochastic optimization technique inspired by swarm intelligence principles, first formalized by Kennedy and Eberhard (1995). The method simulates social behavior observed in biological systems, particularly the collective motion of bird flocks or fish schools, to solve complex optimization problems. Particle Swarm Optimization (PSO) is a computational technique known for its simplicity in implementation and its efficiency in quickly finding viable solutions to complex problems^42^. The process begins with the random selection of initial positions and velocities for each particle within the swarm as illustrated in Eqs. (2), (3). In the subsequent iteration, each particle’s performance is assessed using an objective function, and the best position it has encountered, referred to as the local best ${P}_{best}$, is recorded. When a particle achieves a fitness value that exceeds its previous best, its current position is updated, and the overall best position found by the swarm is designated as the global ${g}_{best}$. In this context, ${x}_{i}$ represents the position of the particle,$D$ denotes the dimensionality of the search space, and ${v}_{i}$ indicates the particle’s velocity, which is usually initialized randomly.

2 $$position:\quad x_{i} = \left( {x_{i1} ,x_{i2} , \ldots ,x_{i} D} \right)$$

3 $$velocity:\quad v_{i} = \left( {v_{i1} ,v_{i2} , \ldots ,v_{i} D} \right)$$

During each iteration, the particles adjust their velocities and positions using Eqs. (4) and (5). If the stopping criteria aren’t met, each particle is reevaluated, and its velocity and position are updated again. The best position a particle has ever reached becomes its new personal best, while the best position found by any particle in the swarm becomes the new global best. This process repeats continuously until the termination conditions are fulfilled^43^

4 $$v_{i} \left( {t + 1} \right) = \omega v_{i} \left( t \right) + c_{1} r_{1} \left[ {P_{best\left( i \right)} - x_{i} \left( t \right)} \right] + c_{2} r_{2} \left[ {g_{best\left( i \right)} - x_{i} \left( t \right)} \right]$$

5 $$x_{i} \left( {t + 1} \right) = x_{i} \left( t \right) + v_{i} \left( {t + 1} \right)$$

where $\omega$ is the inertial weight which determines how much the particle’s previous velocity influences its current movement, ${c}_{1}$ and ${c}_{2}$ are the acceleration coefficients which represent cognitive (individual) and social (group) learning factors, respectively. ${r}_{1}$ and ${r}_{2}$ are the random numbers uniformly distributed between 0 and 1, introducing stochasticity.

**Fig. 7:**
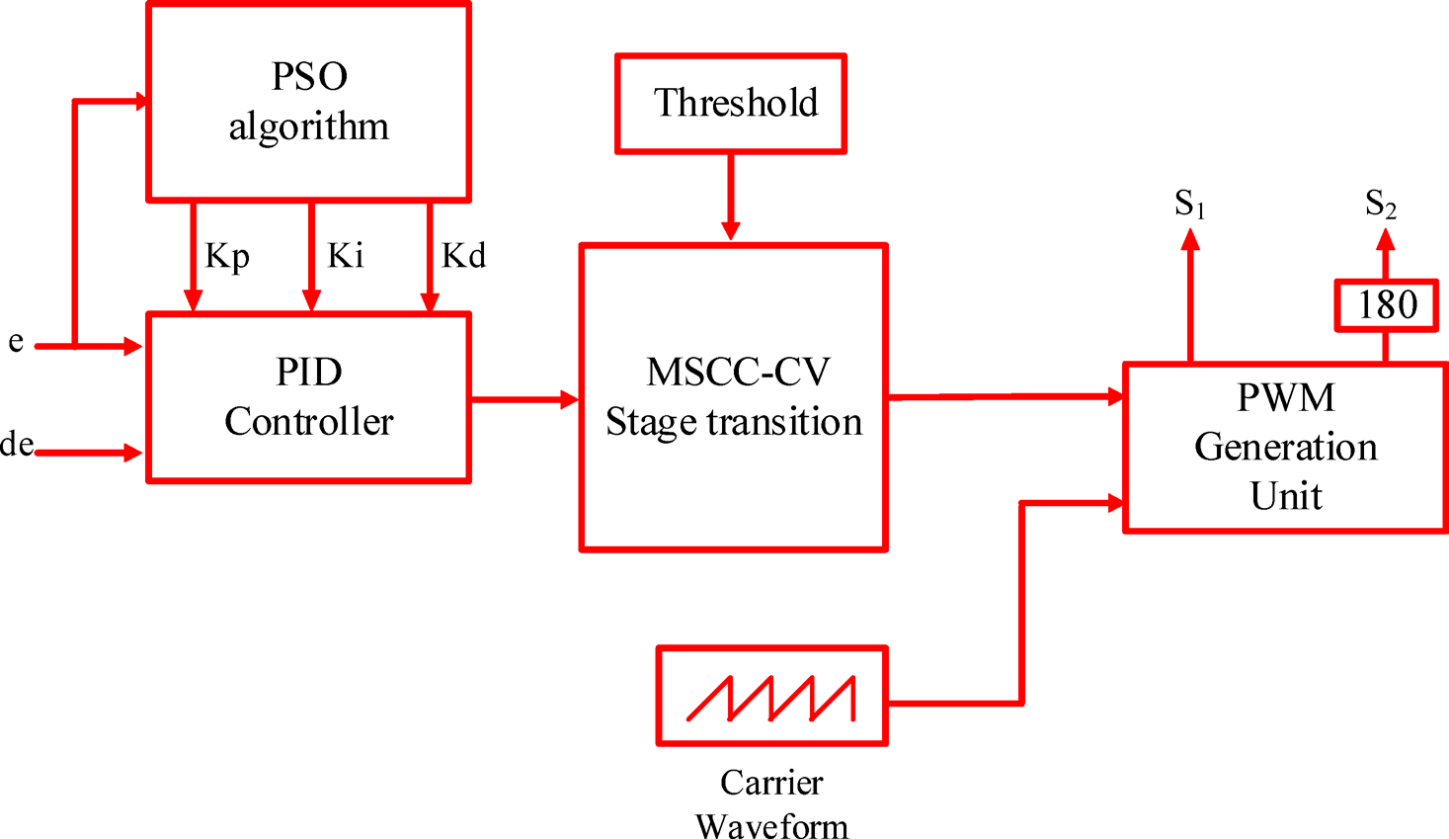
Control scheme for the charging proposed system.

To achieve optimal dynamic performance and control accuracy in the proposed charging system, a particle swarm optimization (PSO) algorithm was employed to continuously fine-tune the PID controller parameters (*K*_*p*_, *K*_*i*_, and *K*_*d*_) through online iteration. The fitness function utilizes the Integral of Time-weighted Absolute Error (ITAE) index, which captures the relationship between error and time. As shown in Eq. (6), a smaller fitness value indicates better control performance. Figure 7 illustrates the structure of the proposed charging control system. The specific goals for performance were set to ensure rapid response times and minimal overshoot.

6 $$J={\int }_{0}^{T}t\left|e(t)\right|dt$$

where e denotes the system error, and T denotes the total system runtime optimization.

Since the PID controller requires tuning three parameters (*K*_*p*_, *K*_*i*_, and *K*_*d*_), the particle swarm optimization (PSO) algorithm operates in a three-dimensional search space. Through multiple simulation tests, the optimal PID parameters were determined and are listed in Table 5^44^. The detailed steps for tuning the PID controller using the particle swarm optimization (PSO) algorithm are outlined in the pseudocode provided in Table 6^45^.

**Fig. 8:**
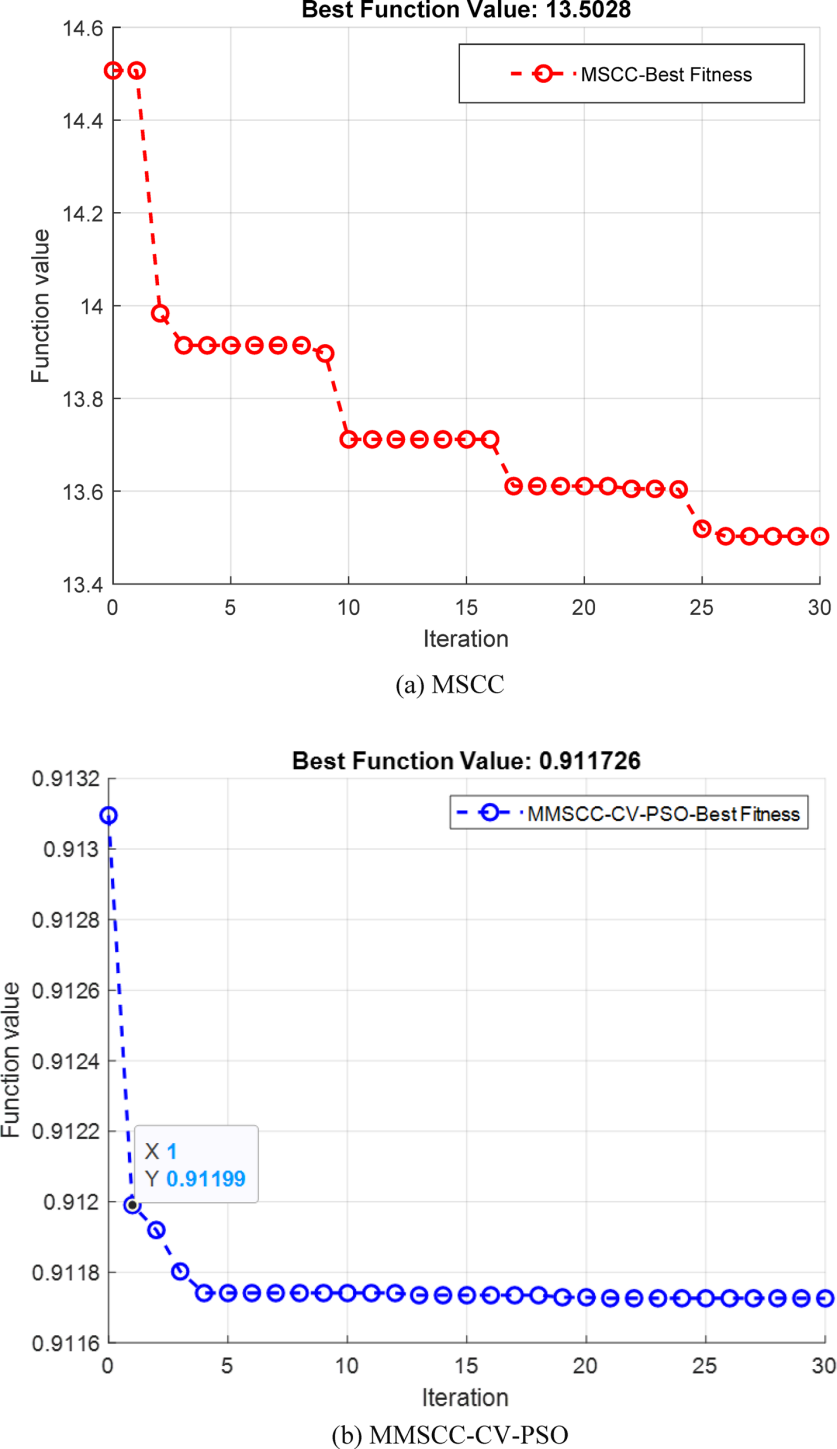
PSO convergence curves for (**a**) MSCC and (**b**) MMSCC-CV-PSO. Both methods were optimized under identical PSO parameters and termination criteria.

The MSCC and MMSCC controllers were optimized using the same PSO–PID framework to ensure a fair comparison. The PSO objective minimized charging time while constraining temperature rise and current overshoot. Optimization was terminated either when the maximum number of iterations (n_max_ = 30) was reached or when the improvement in the global best cost between successive iterations fell below 0.001. The full set of PSO parameters are reported in Table 5. The final optimized (Kp, Ki, and Kd) values are reported in Table 7. Figure 8 illustrates the PSO convergence behavior, where the best objective value decreases monotonically and eventually stabilizes, confirming that both controllers were tuned to convergence under identical conditions.the MMSCC-CV-PSO controller converged to a lower objective value, consistent with its superior performance in charging time reduction, temperature rise mitigation, and SOC tracking accuracy.

**Table 5:** Optimized parameter configuration for particle swarm optimization algorithm.

Parameter	Value
Swarm population $\left( {np} \right)$	40
Termination criterion $\left( {n_{max} } \right)$	30
Dynamic inertia coefficients (ω_min_, ω_max_)	0.4,0.9
Lower band $\left( {LB} \right)$	0
Upper band $\left( {UB} \right)$	1000
Learning coefficients (c_1_, c_2_)	0.8, 0.8
Randomization r_1_, r_2_	0.2,0.3
Constriction factor m	2
Fitness evaluation metrics (γ_1_, γ_2_)	0.5, 0.5

**Table 6:**
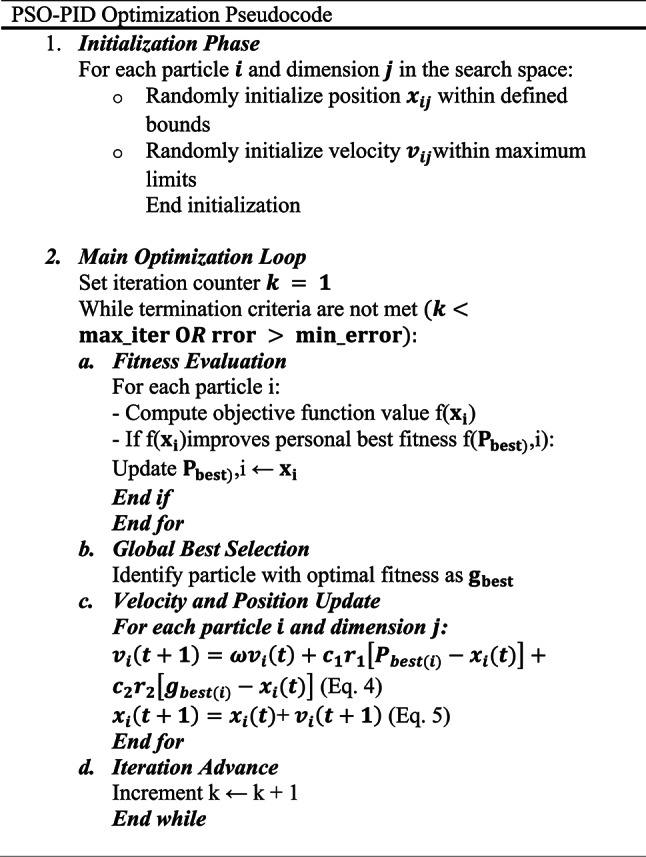
PSO-PID optimization pseudocode.

**Table 7:** Final optimized controller gains for MSCC and MMSCC-CV-PSO obtained from the PSO–PID framework.

Method	Kp	Ki	Kd
MSCC	1000	$19.532{e}^{2}$	0
MMSCC-CV-PSO	5.986	$6.886{e}^{2}$	0.01

## Simulation methodology and comparative analysis

To evaluate the effectiveness of our proposal, we conduct numerical simulation experiments in this section. First, we outline the criteria for assessment. Next, we explain the assessment process, followed by presenting the simulation results and comparisons.

### Performance evaluation metrics

The comparative analysis examined four key parameters:

1. *Charging dynamics* Current/voltage temporal characteristics
2. *Thermal performance* Peak heat generation and thermal gradients
3. *Charging efficiency* Time-to-100%-SoC
4. *Charge completeness* Maximum achievable SoC

### Experimental setup

A series of experiments are implemented in the MATLAB/Simulink® tool to validate our proposed algorithms comprehensively. The experiments are carried out in an Intel Core i7 dual-core CPU with a frequency of 2.9 GHz, 16 GB of memory, and a Windows 10 operating system. The test platform comprised:

1. Lithium ion battery model.
2. Three charging controller implementations:
  - CC–CV (Constant Current–Constant Voltage)
  - Optimized MSCC (Multistage CC)
  - Proposed MMSCC-CV-PSO
3. Precision measurement blocks for:
  - Terminal voltage (± 0.1% accuracy)
  - Heat generation (± 0.5 °C resolution)
4. State-of-Charge (Coulomb counting with < 1% error)

To ensure a fair comparison among the three charging strategies CC–CV, Optimized MSCC, and MMSCC-CV-PSO critical simulation parameters were kept identical across all scenarios. These include the initial state of charge (SOC), maximum voltage ($Vmax$), and cutoff current, ensuring consistency in starting conditions and termination criteria. Table 7 summarizes these parameters alongside a brief description of each charging method. The proposed MMSCC-CV-PSO method is evaluated against traditional CC–CV and Standard MSCC approaches. The Optimized MSCC parameters were selected following references^11^ and^33^ (Section “Conventional multi-stepped constant current (MSCC) charging”) with moderate tuning to ensure realistic and safe operation, without applying global optimization. This ensures a fair and representative comparison. The results demonstrate that MMSCC-CV-PSO achieves faster charging, reduced heat generation, and improved state-of-charge (SOC) accuracy compared to both benchmarks, highlighting its practical benefits under realistic conditions.

As shown in Table 8, all methods share the same initial SOC, maximum voltage, and cutoff current settings. This uniformity ensures that performance differences are directly attributed to the control strategies themselves. The proposed MMSCC–CV–PSO method introduces a novel optimization approach that improves charging efficiency and reduces stress on the battery.

**Table 8:** Simulation setup for the three charging strategies.

Method	Initial state of charge (SOC)	Maximum voltage ($Vmax$)	Cutoff current
CC-CV	0	29.4	1.9
Optimized MSCC	0	29.4	1.9
MMSCC-CV-PSO	0	29.4	1.9

### Results and discussion

**Fig. 9:**
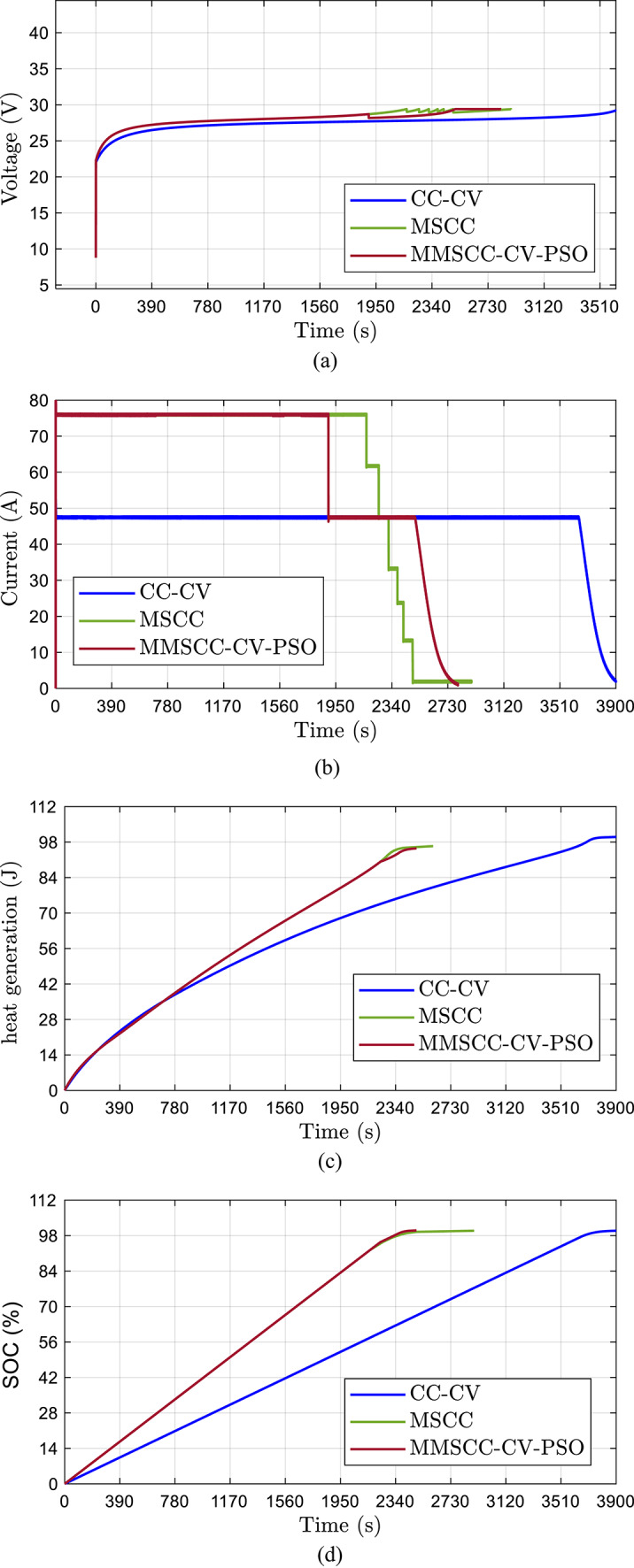
Comparisons between CC–CV, Optimized MSCC, and proposed MMSCC-CV-PSO: (**a**) Battery voltage (**b**) Battery current (**c**) heat generation (**d**) Battery SOC.

The proposed MMSCC-CV-PSO charging strategy was evaluated and compared against two conventional techniques—namely the Constant Current–Constant Voltage (CC–CV) method and the optimized Multi-Stepped Constant Current (MSCC) approach. The performance of each method was assessed based on key metrics such as terminal voltage behavior, current profile, heat generation, state of charge (SOC) evolution, and overall energy efficiency. The results, illustrated in Fig. 9a–d and Table 8, provide a comprehensive understanding of the enhancements achieved by the proposed technique.

As illustrated in Fig. 9a, the CC–CV method features a straightforward charging profile: a constant current phase followed by a shift to a fixed voltage plateau at 29.4 V. While this approach is simple and widely used, it lacks flexibility in adapting to battery dynamics during charging. Conversely, the optimized MSCC method adopts a stepped voltage profile, allowing more granular control over the charging process. The proposed MMSCC-CV-PSO method builds on this by introducing two constant-current (CC) phases followed by a constant-voltage (CV) phase. This multi-level voltage regulation enables the system to respond dynamically to battery needs, reducing electrical stress and potentially improving cell longevity.

The analysis of current profile dynamics and behavior further emphasizes the adaptability of the MMSCC-CV-PSO method as shown in Fig. 9b. In the CC–CV method, current decays exponentially during the constant voltage phase—a process that can be inefficient in later charging stages. The optimized MSCC method utilizes a sequence of constant current steps with fixed durations, offering improved consistency but limited responsiveness. The MMSCC-CV-PSO, however, features a hybrid approach: it divides the current profile into two major dynamic phases. This enables real-time current adjustment based on the battery’s state, enhancing control over charge flow and contributing to reduced energy losses and better thermal behavior.

Figure 9c presents the heat generation data during the charging process. The CC–CV method results in the highest thermal output, with peak heat generation reaching 100 J. This level of heat can accelerate battery degradation and limit lifespan. The MSCC method shows modest improvement with a reduced peak of 96.5 J. The MMSCC-CV-PSO approach achieves further enhancement by capping the maximum heat at 95.5 J, reflecting a 1.04% improvement over MSCC and 4.5% over CC–CV. These results underscore the method’s effectiveness in thermal regulation, which is crucial for maintaining safe and stable battery operation.

SOC analysis shown in Fig. 9d reveals clear advantages of the proposed method in terms of charging speed and completeness. The MMSCC-CV-PSO method achieves full SOC (100%) in just 41.3 min, significantly faster than the optimized MSCC (48.1 min) and CC–CV (60 min) methods. Additionally, it reaches 99% SOC approximately 29.5% faster than the CC–CV method, demonstrating its capability to deliver a quicker charge without compromising energy efficiency. This acceleration in charging time can be particularly valuable for time-sensitive energy storage applications.

Table 9 summarizes the performance metrics across the three conducted methods. These quantitative results clearly establish the superiority of the MMSCC-CV-PSO method. It consistently outperforms conventional techniques across all critical metrics, including thermal performance, charge time, SOC accuracy, and energy efficiency.

**Table 9:** Quantifies the performance advantages.

Performance metric	CC-CV	Optimized MSCC	MMSCC-CV-PSO	Observed improvement
Charge time (min)	60	48.1	41.3	15% faster compared to MSCC
Maximum heat generation (J)	100	96.5	95.5	1.04% lower than MSCC
Final SOC (%)	99.87	99.97	100	+ 0.03 percentage points vs. MSCC
Energy efficiency (%)	98.5	99	99.6	+ 0.6 percentage points versus MSCC

**Fig. 10:**
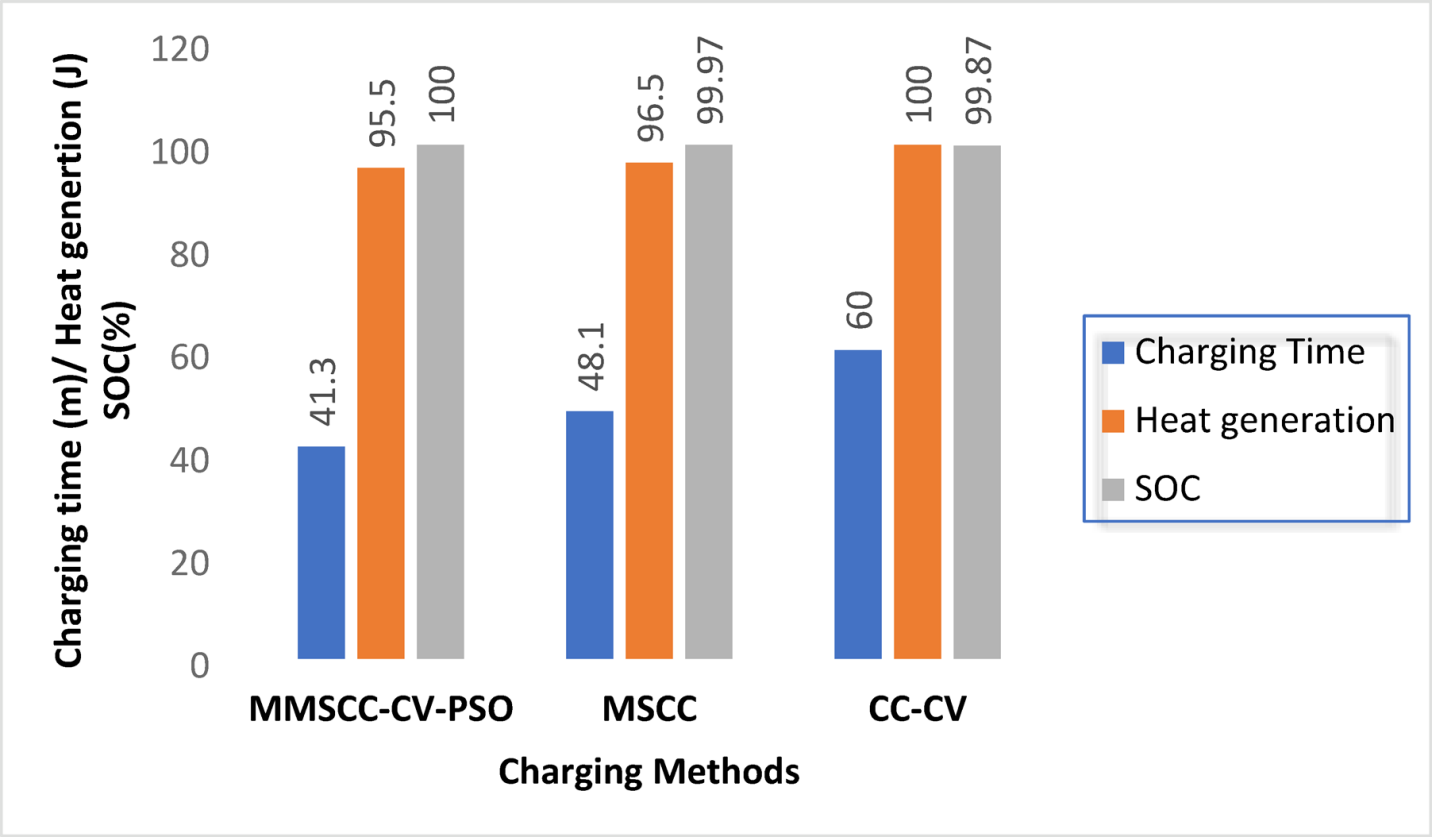
Performance comparison of the simulation results.

Finally, Fig. 10 presents a consolidated graphical comparison, highlighting the key simulation results for each method. The visual synthesis clearly demonstrates the enhancements offered by MMSCC-CV-PSO, validating its capability as an effective and efficient battery charging strategy. The method’s ability to combine fast charging, thermal safety, and high energy efficiency makes it a strong candidate for advanced lithium-ion battery management systems.

### Impact on battery life extension

The experimental results demonstrate that the proposed multi-step fast charging strategy not only shortens the total charging time but also significantly reduces heat generation compared to conventional fast-charging profiles. Elevated cell temperature is a key accelerator of degradation mechanisms such as lithium plating, excessive SEI layer growth, and mechanical stress on active materials, all of which contribute to rapid capacity fade. By maintaining a lower thermal profile throughout the charging process, the proposed method effectively suppresses these side reactions, thereby slowing the rate of performance decay. Furthermore, controlled current transitions between charging steps reduce electrode overpotential, minimizing the risk of lithium plating even at higher charging rates. These combined effects indicate that the method can extend the battery’s service life while maintaining high charging efficiency.

## Conclusion and future work

This study presents an advanced and optimized charging methodology—Modified Multi-Stepped Constant Current–Constant Voltage integrated with Particle Swarm Optimization (MMSCC-CV-PSO)—designed to enhance the performance of lithium-ion battery energy storage systems. Unlike conventional techniques such as the standard MSCC and CC–CV methods, the proposed strategy dynamically adjusts the charging profile using an intelligent optimization algorithm to minimize heat generation and improve energy transfer efficiency. Simulation results demonstrate that the MMSCC-CV-PSO approach significantly reduces peak thermal output during charging, which directly contributes to minimizing thermal stress on battery components and thus extends the battery’s operational lifespan. Furthermore, the method reliably achieves a full state of charge (100% SOC), slightly surpassing conventional approaches and ensuring optimal utilization of battery capacity without compromising safety. These performance gains are achieved while maintaining system stability and thermal safety margins, positioning the MMSCC-CV-PSO technique as a robust and effective solution for modern energy storage applications. The findings highlight the approach’s potential to support the growing demand for fast, efficient, and thermally controlled charging in electric vehicles and other battery-powered technologies.

Despite these promising results, the current validation is limited to simulations with a fresh Li-ion model and does not account for degradation mechanisms such as capacity fade, internal resistance growth, or lithium plating. As part of our future work, we plan to extend this study by implementing the proposed strategy in a hardware-in-the-loop (HIL) platform and conducting experiments on real Li-ion cells. In addition, extended cycle-life testing will be performed to evaluate the method’s impact on battery State of Health (SoH). Further improvements will also focus on enhancing the adaptability of the controller through online re-optimization or adaptive schemes to account for parameter drift caused by aging. Finally, benchmarking the PSO-based optimization against other metaheuristic algorithms will be considered to further validate its robustness and competitiveness.

## Data Availability

The datasets used and/or analysed during the current study available from the corresponding author on reasonable request.
